# Development of the novel Pictor PictVet™ *Mycoplasma bovis* IgG multiplex ELISA for the detection of *Mycoplasma bovis* infections in cattle

**DOI:** 10.3389/fvets.2025.1664919

**Published:** 2025-09-10

**Authors:** Yoichi Furuya, Piyush Bugde, Bhoopika Shetty, Farina Nor Hashimi, Hamideh Gholizadeh, Dave Whittaker, Jimena Tejerina, Natasha Gordon, Kelly A. Tivendale, Nadeeka K. Wawegama, Glenn F. Browning, Andrea Kinga

**Affiliations:** ^1^Pictor Limited, Auckland, New Zealand; ^2^Asia-Pacific Centre for Animal Health, Melbourne Veterinary School, Faculty of Science, The University of Melbourne, Parkville, VIC, Australia

**Keywords:** *Mycoplasma bovis*, multiplex, ELISA, immunoassay, antibodies

## Abstract

Early and accurate detection of infection with *Mycoplasma bovis (M. bovis)* is critical for managing disease caused by this pathogen, particularly in eradication campaigns, such as New Zealand’s National *M. bovis* Eradication Programme. In response to the launch of the eradication programme, we developed and evaluated a novel multiplex ELISA assay—the Pictor PictVet™ *M. bovis* IgG Multiplex ELISA. Two *M. bovis* antigens, MilA and K310, were incorporated into the Pictor PictVet™ *M. bovis* Multiplex ELISA to detect *M. bovis*-specific serum IgG. Studies were conducted to determine the assay agreement with the ID Screen® *Mycoplasma bovis* Indirect ELISA (IDvet). A collection of sera from New Zealand cattle, previously characterized as IDvet-positive (+) and IDvet-negative (−), was used for these evaluations. Binding to the two different *M. bovis* antigens, MilA and K310, had high estimates of agreement, with PPAs of 89.3 and 96.4%, and a NPA of 99.2%. When the results from both the MilA and K310 components of the assay were combined to determine the assay outcome, the PPA increased to 100%, while the NPA remained high, at 98.4%, with an overall agreement of 98.9%. Multiplexing of two *M. bovis* antigens enhanced the diagnostic performance of the indirect ELISA in detecting *M. bovis*-specific IgG in serum samples. These findings suggest that the Pictor PictVet™ *M. bovis* IgG Multiplex ELISA could be a valuable tool for early detection and surveillance for infection with *M. bovis* in cattle.

## 1 Introduction

*Mycoplasma bovis* (*M. bovis*) is a significant bacterial pathogen of cattle, causing a variety of diseases that have severe impacts on animal health and agricultural profitability. It was first identified in the United States in the 1960s and has since spread to most major cattle-producing countries (1). As a member of the class Mollicutes, *M. bovis* is characterized by its small genome, lack of a cell wall, and ability to persist in host tissues. These features enable *M. bovis* to evade immune responses and render it inherently resistant to antibiotics that target cell wall synthesis (2, 3). It can cause respiratory disease, mastitis, arthritis, middle ear infections and reproductive tract disease in cattle, resulting in substantial economic losses in dairy and beef industries worldwide (4–6). Transmission occurs through close contact with infected animals, and from contaminated environments and asymptomatic carriers within herds, complicating efforts to prevent and control outbreaks (7, 8).

The incursion of *M. bovis* into New Zealand was first recognised in 2017—a significant event, as it was one of the last major cattle-rearing countries free of this pathogen (9). In response, New Zealand launched the world’s first *M. bovis* eradication programme, an initiative aimed at eliminating the pathogen from the national herd. This unprecedented eradication effort demanded accurate diagnostic testing to facilitate early detection and effective disease management. However, current diagnostic testing for infection with *M. bovis* faces several challenges, particularly in terms of sensitivity and specificity. Although polymerase chain reaction (PCR) assays are widely used to directly detect *M. bovis* DNA in various sample types, they have limitations that reduce their efficacy in the field. The intermittent shedding of *M. bovis* by infected animals may result in false-negative results, particularly from nasal swabs or milk samples (10). Furthermore, the costs associated with routine PCR testing of large numbers of animals in a national herd are significant, potentially straining resources in a large-scale eradication programme. Therefore, PCR is primarily used as a confirmatory test for ELISA positive samples (11). These limitations create a diagnostic gap, particularly in monitoring the effectiveness of eradication programmes, where reliable and early detection of infection with *M. bovis* at low levels is critical. In light of these challenges, our study focused on the development of a robust ELISA test to address these diagnostic shortcomings, providing a more consistent and reliable method for tracking *M. bovis* infections in New Zealand. We aimed to assess the analytical performance of the Pictor PictVet™ *M. bovis* IgG Multiplex ELISA, which incorporates two *M. bovis* antigens, MilA and K310.

## 2 Method and materials

### 2.1 Serum samples

To develop and validate the Pictor PictVet™ *Mycoplasma bovis* Multiplex ELISA serum assay, we used a total of 180 heat-treated bovine serum samples, previously characterized using the commercially available ID Screen® *Mycoplasma bovis* Indirect ELISA (IDvet), which served as the reference test and was performed according to the manufacturer’s instructions. Fifty-six of these samples were collected in late 2022 from spring-calving herds located on a South Island farm in New Zealand. The cohort included both male and female Jersey and Friesian cattle spanning a range of ages, from calves to adults. Animals were sampled during routine surveillance testing under the National *M. bovis* Eradication Programme. All animals appeared clinically healthy at the time of collection, suggesting subclinical infections. These 56 samples tested positive on the IDvet ELISA and were subsequently evaluated on Pictor’s multiplex platform between January and February 2023. The remaining 124 samples, which tested negative on the IDvet assay, were randomly selected from archived serum specimens. These samples were sourced from *M. bovis*-free herds across various geographic regions in New Zealand. The cattle represented a mix of sexes and ages, similar to the positive cohort. Both collection and testing of these samples occurred in January 2023.

All samples were acquired via Assure Quality, a New Zealand government-owned national laboratory involved in diagnostic testing under the National *M. bovis* Eradication Programme. The samples were stored at the Physical Containment Level 2 (PC2) facility of SVS Laboratories in Hamilton, New Zealand. All serum samples were heat-treated and stored at −80 °C until testing to preserve antibody integrity and mitigate any risk of antibody decay. The timeframe between sample collection and testing ranged from 1 to 2 months for both cohorts, ensuring minimal degradation and maintaining sample quality for accurate assay performance evaluation.

The laboratory staff conducting the Pictor PictVet™ ELISA were not formally blinded to the IDvet comparator test results during assay evaluation.

### 2.2 *Mycoplasma bovis* antigens

The assay incorporated two distinct surface proteins of *M. bovis* – MilA (*MBOVPG45_0710*), which has previously been shown to be useful as the basis for highly sensitive and specific conventional indirect ELISAs (12–18), and a novel antigen, K310 (*MBOVGP45_310*). Recombinant glutathione S-transferase (GST) fusion proteins were expressed in and purified from *E. coli* carrying pGEX expression plasmids encoding the mature *M. bovis* MilA and *M. bovis* K310 proteins (17). The manufacturer (GenScript) confirmed the identity of the proteins by SDS-PAGE and Western blotting using anti-GST monoclonal antibodies (mAb). The identity of each of the proteins was further confirmed using antigen-specific monoclonal antibodies in the Pictor PictVet™ *M. bovis* Multiplex ELISA.

### 2.3 *Mycoplasma bovis* specific monoclonal antibodies

Monoclonal antibodies specific to *Mycoplasma bovis* antigens MilA and K310 were custom-developed by GenScript. Purified MilA and K310 antigens, expressed in *E. coli* BL21 Star™ (DE3) after cloning into the pGEX-4 T-1 vector, were used to immunize New Zealand White rabbits up to three times. Peripheral blood mononuclear cells were then isolated, and antigen-specific B cells were identified and selected. The variable regions of the selected monoclonal antibodies (VH and VL) were sequenced and retained. These rabbit-derived variable regions were genetically fused to bovine IgG constant regions (CH and CL) to generate rabbit–bovine chimeric antibodies, ensuring compatibility with bovine-specific secondary detection antibodies. Rabbit VH/VL and bovine CH/CL sequences were cloned into pcDNA3.4 expression vectors incorporating optimized signal peptides and Kozak consensus sequences. These constructs were co-transfected into Chinese Hamster Ovary (CHO) cells using GenScript’s TurboCHO™ system, employing an optimized heavy-chain to light-chain plasmid ratio to maximize antibody expression. Recombinant antibodies were produced in CHO cells, and the purified supernatant was used for assay development and specificity testing. Anti-bovine IgG secondary antibodies targeting the Fc region were used to avoid interference with antigen-binding domains during detection.

### 2.4 Pictor PictVet™ *Mycoplasma bovis* multiplex ELISA serum assay

**Figure 1:**
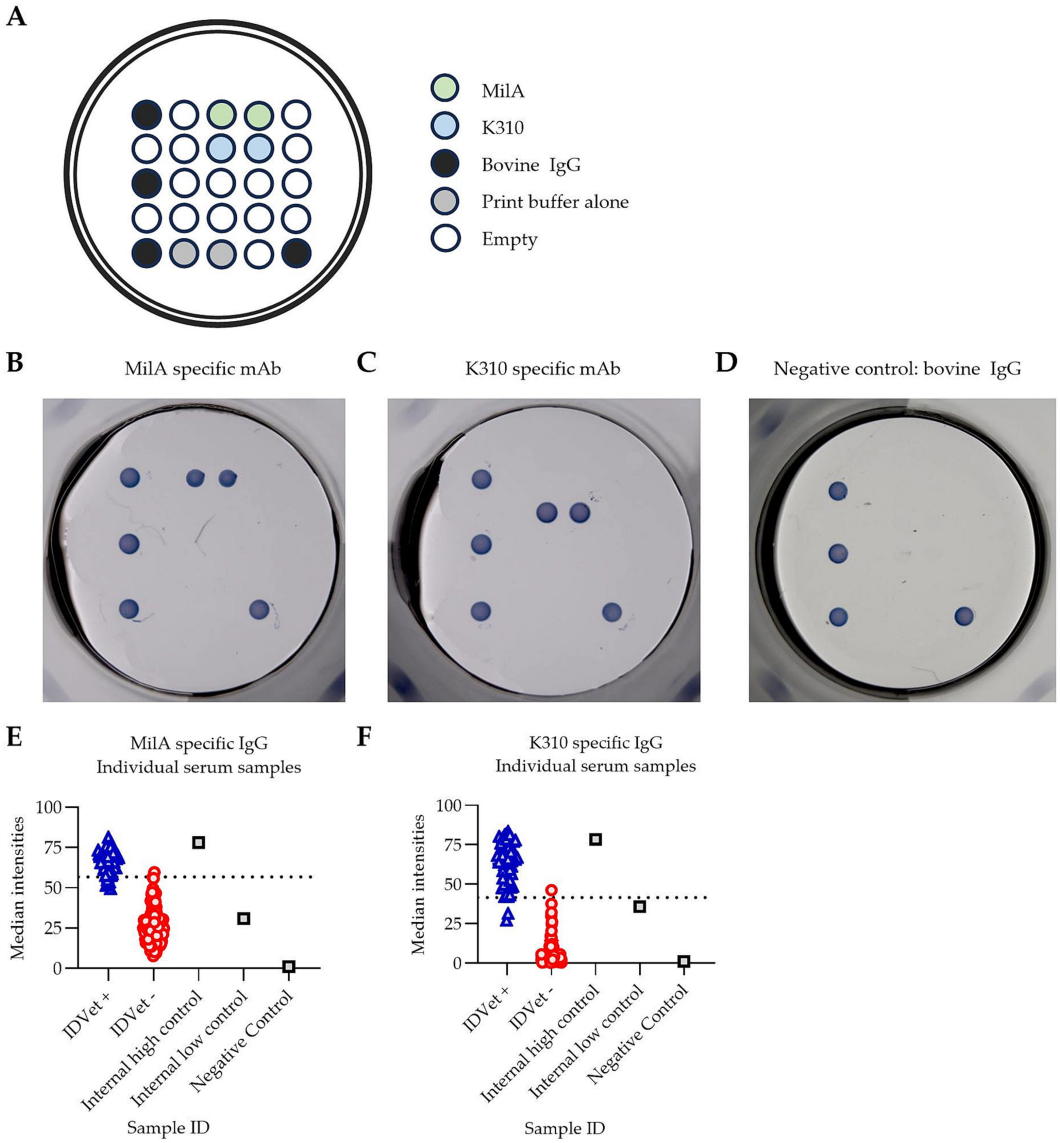
Pictor PictVet™ *Mycoplasma bovis* Multiplex ELISA detects *M. bovis* specific IgG antibodies. **(A)** The layout of the Pictor PictVet™ *Mycoplasma bovis* Multiplex ELISA plate, showing two *M. bovis* antigens, MilA and K310. Bovine IgG and print buffer were also included in the panel as internal controls. A representative image of the assay using mAbs with bovine constant regions, specific for **(B)** MilA and **(C)** K310. **(D)** A negative control sample, in which no *M. bovis*-specific antibodies were present. The absence of stained spots, other than for the bovine IgG internal positive control, indicates the absence of specific binding in these wells. Scatter plots showing the spread of signal intensities for **(E)** the MilA spots and **(F)** the K310 spots when IDvet-positive and IDvet-negative serum samples were assayed. A total of 56 IDvet-positive and 124 IDvet-negative serum samples were tested. The dotted lines indicate the assay’s cut-offs.

PictArray™ Multiplex ELISA plates were prepared by dispensing 10 nL of each of the *M. bovis* antigens, MilA and K310, onto a 96-well ELISA plate, as shown in Figure 1A. Additionally, bovine IgG was included as part of the panel to serve as an internal positive control. A print buffer control was included to demonstrate the background signal intensity due to the print buffer and/or antigen carry over. The spots were printed in duplicate. PictArray™ Multiplex ELISA plates were then incubated with a blocking solution to reduce non-specific binding of antibodies to the ELISA plate.

A conventional indirect ELISA was performed using PictArray™ Multiplex ELISA plates. Briefly, 100 μL of a 1:100 dilution of each of the serum samples was added to each of the wells and the plate incubated at 37 °C for 30 min. The plates were then washed 5 times. After washing, 100 μL of horseradish peroxidase-conjugated sheep anti-bovine IgG heavy and light chain antibody was added and the plate was incubated at 37 °C for 30 min. The plates were washed 5 times, and 100 μL of TMB substrate was added and the plate incubated at room temperature for 20 min. The plates were then read using the PictImager™ (sciREADER CL2 colorimetric plate reader), which captures high-resolution images of the spots. The spot signal intensities were then measured using Pictorial™ software.

### 2.5 Positive and negative agreement

As the ID Screen® *Mycoplasma bovis* Indirect ELISA (IDvet) was used as a comparator test, positive agreement, negative agreement, and overall agreement were calculated to assess concordance with the comparator test. The positive percent agreement (PPA) was defined as 100% x a / (a + c), and the negative percent agreement (NPA) as 100% x d / (b + d), and overall agreement as 100% x (a + d) / (a + b + c + d), where a = the number of samples positive in both tests, b = the number of samples positive in the PictVet test but negative in the IDvet test, c = the number of samples negative in the PictVet test but positive in the IDvet test, and d = the number of samples negative in both tests. These metrics were calculated to evaluate the test’s diagnostic performance in the absence of a recognised reference standard.

### 2.6 Analytical sensitivity

The limit of detection (LoD) was determined empirically following the CLSI EP17-A guidelines (19, 20). First, the Limit of Blank (LoB) was established by analyzing blank samples and determining the 95th percentile of the blank signal distribution. To estimate the LoD, a low concentration sample was measured in 20 independent replicates over three non-consecutive days. The LoD was defined as the lowest concentration at which ≥95% (at least 19 out of 20) of the replicate measurements produced a signal greater than the LoB. This approach does not assume a normal distribution. The LoD was determined using two monoclonal antibodies, one specific for MilA and one specific for K310. Based on initial two-fold serial dilutions, three concentrations close to the LoB were selected for the analytical sensitivity study. Each of the three concentrations was assayed in 20 replicates, and the LoD was defined as the concentration at which 95% of the 20 samples had a signal intensity greater than the LoB. This assay was repeated three times on different days.

### 2.7 Data interpretation

Pictor PictVet™ *Mycoplasma bovis* Multiplex ELISA results were reported both as quantitative spot signal intensities and as qualitative outcomes (positive or negative). The assay includes two antigens, MilA and K310—each printed as a discrete spot within the same well. Cut-off values were determined based on the median spot colour intensity (unitless values generated by image analysis software) and set at 56.75 for MilA and 41.59 for K310. A sample was considered positive if the median spot intensity exceeded the cut-off for either antigen. If both values fell below their respective thresholds, the sample was interpreted as negative.

### 2.8 Descriptive statistics

Analyses were performed using GraphPad Prism version 10.5.0 (GraphPad Software, San Diego, CA). The software was used to calculate summary statistics such as means, standard deviations, and confidence intervals, where applicable.

## 3 Results

### 3.1 Pictor PictVet™ *Mycoplasma bovis* multiplex ELISA exhibited a high diagnostic performance

To develop a highly sensitive and specific assay for screening for antibodies against *M. bovis*, we designed a multiplex ELISA assay, incorporating two different *M. bovis* antigens, MilA and K310, using Pictor’s multiplex ELISA platform (Figure 1A). The figure shows where each antigen was dispensed into each well of a 96-well ELISA plate to create the multiplex *M. bovis* test panel. Specific binding was visualized as a stained spot on the multiplex ELISA plate. We hypothesized that incorporating two distinct *M. bovis* antigens would improve the assay performance, as the use of two antigens was likely to increase the likelihood of detecting *M. bovis*-specific IgG antibodies in each animal, as antibody responses against two distinct surface proteins of *M. bovis* could be detectable in each sample. To confirm the specificity of the Pictor PictVet™ *Mycoplasma bovis* Multiplex ELISA, monoclonal antibodies against MilA (Figure 1B) and K310 (Figure 1C) were first used to validate the presence of these antigens on the assay platform. Polyclonal bovine IgG spots were also included to demonstrate the absence of any specific binding in wells where no *M. bovis*-specific antibodies were present (Figure 1D). This negative control established the baseline for background signal intensity, ensuring that any signal detected in the assay was due to specific antigen–antibody interactions and not due to non-specific binding.

To test our hypothesis, the diagnostic performance of the Pictor PictVet™ *Mycoplasma bovis* Multiplex ELISA was compared to the IDvet ELISA kit. There was a distinct separation between the IDvet+ and IDvet- samples when the results from assaying them in the Pictor PictVet™ *Mycoplasma bovis* Multiplex ELISA were plotted based on spot signal intensities (Figures 1E,F). Reactions with both the MilA and the K310 antigens in the assay differentiated between positive and negative serum samples. The positive samples had higher signal intensities for both the MilA and K310 antigens than the negative samples. The well-defined separation of these two groups indicated that the assay could distinguish between infected and uninfected animals.

The diagnostic performance of the Pictor PictVet™ *Mycoplasma bovis* Multiplex ELISA was evaluated by comparing it to the commercially available IDvet ELISA, which served as the comparator method (Table 1). A total of 180 serum samples were tested. The Pictor PictVet™ ELISA correctly identified all 56 IDvet-positive samples (PPA of 100%) and 122 of 124 of the IDvet-negative samples (NPA of 98.4%), yielding an overall agreement of 98.9%. These results demonstrated strong agreement with the IDvet ELISA and support the potential of the Pictor PictVet™ Multiplex ELISA as a reliable alternative for detecting antibodies against *M. bovis* in cattle.

**Table 1:** Agreement between the Pictor PictVet™ Multiplex ELISA and the IDvet ELISA.

		Comparator method (IDvet)		Total
		+	−	
Pictor PictVet™ *Mycoplasma bovis* Multiplex ELISA	+	56	2	58
	−	0	122	122
Total		56	124	180

To assess individual antigen performance within the Pictor PictVet™ *Mycoplasma bovis* Multiplex ELISA, the two target antigens, MilA and K310, were evaluated using the IDvet ELISA as the comparator test (Table 2). The MilA antigen alone identified 50 of 56 positive samples (PPA of 89.3%), with only one false positive (NPA of 99.2%). The K310 antigen alone identified 54 of 56 positive samples (PPA of 96.4%) with only one false negative (NPA of 99.2%). However, the MilA/K310 combination yielded a higher overall performance, detecting all 56 positive samples (PPA of 100%), with only two false positives (NPA of 98.4%). These findings indicated that, while both individual antigens perform well, the MilA/K310 combination provides the best diagnostic accuracy for the detection of *Mycoplasma bovis* specific antibodies, supporting the inclusion of both antigens as core components of the multiplex assay.

**Table 2:** Multiplexing the two antigens improved the performance of the assay.^a^

Antigen	False +	True +	False −	True −	Total	% PPA	% NPA
MilA/K310	2	56	0	122	180	100	98.4
MilA	1	50	6	123	180	89.3	99.2
K310	1	54	2	123	180	96.4	99.2

a Positive percent agreement and negative percent agreement were calculated for the multiplex assay (MilA / K310) and the individual antigens (MilA and K310) separately yielding an overall agreement of 98.9%.

### 3.2 Pictor PictVet™ *Mycoplasma bovis* multiplex ELISA exhibits superior analytical sensitivity compared to the IDvet ELISA

**Figure 2:**
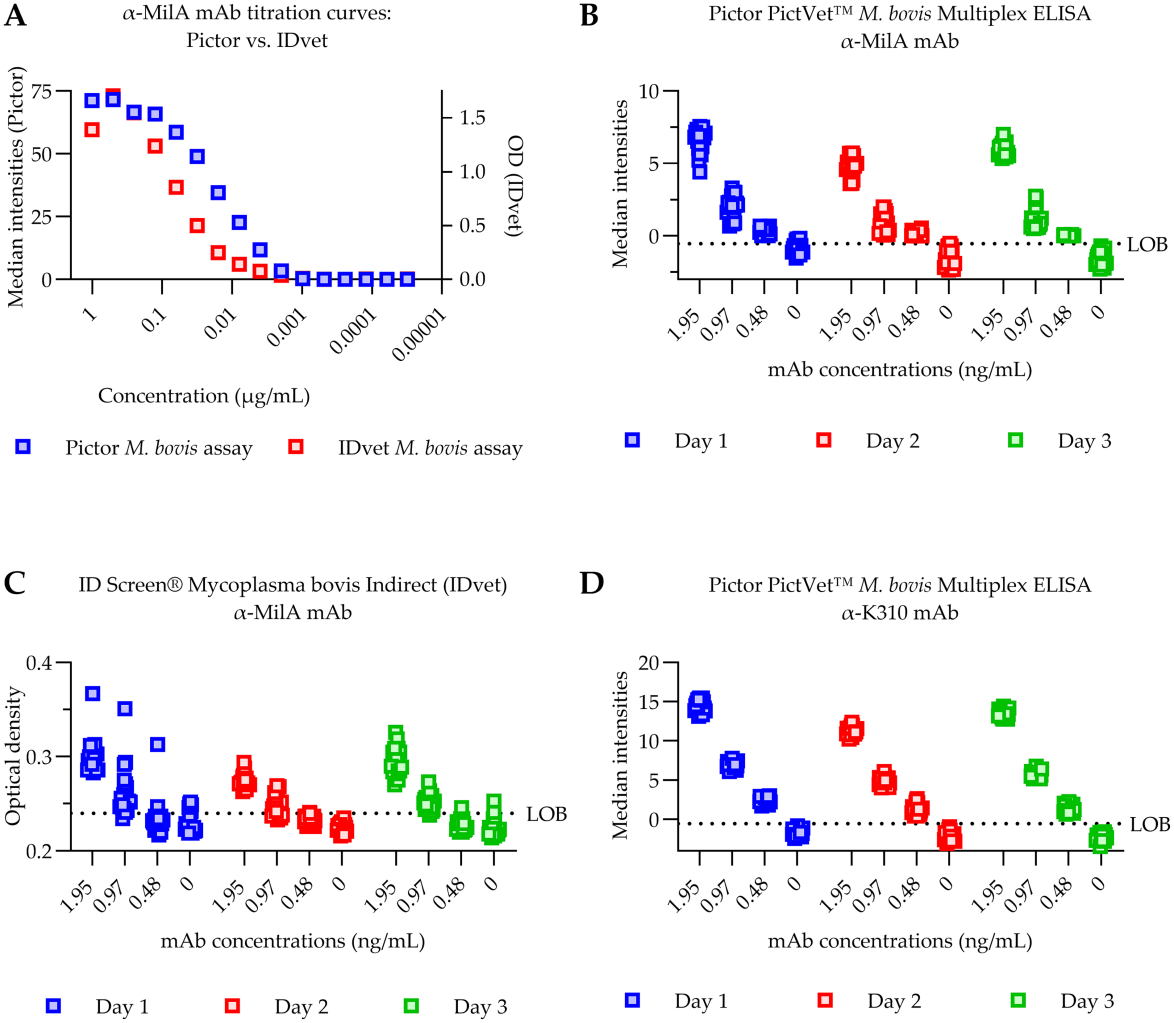
The Pictor PictVet™ Multiplex ELISA had superior sensitivity to the IDvet ELISA in detecting bovinised monoclonal antibodies against the *M. bovis* MilA protein. **(A)** Titration curves of bovinised monoclonal antibodies against MilA antigen measured in the Pictor and IDvet assays. The Pictor ELISA results are plotted as median spot intensities (left axis) and the IDvet results are plotted as optical density (OD) values (right axis). **(B–D)** Determination of the LoD for the MilA and K310 antigens. Twenty replicate measurements were performed at mAb concentrations near the LoB and assayed across three independent days - Day 1 (blue), Day 2 (red), and Day 3 (green). **(B)** The Pictor PictVet™ ELISA results with the anti-MilA mAb. **(C)** The IDvet ELISA results with the anti-MilA mAb. **(D)** The Pictor PictVet™ ELISA results with the anti-K310 bovine mAb. The LoD was defined as the lowest concentration at which ≥95% of the 20 replicates yielded a signal above the LoB across all three assay runs. Dashed lines indicate the LoB for each assay.

To compare the analytical sensitivity of the Pictor PictVet™ *Mycoplasma bovis* Multiplex ELISA with the commercially available IDvet ELISA, monoclonal antibody (mAb) titration experiments were performed targeting the MilA and K310 antigens. As shown in Figure 2A, both assays yielded similar titration curves across a wide range of anti-MilA mAb concentrations. However, at higher antibody concentrations, particularly at 1 μg/mL, a decreased signal was seen in the IDvet ELISA compared to that seen at 0.5 μg/mL, suggesting a possible prozone effect. In contrast, in the Pictor PictVet™ ELISA, the signal was saturated at 1 μg/mL, but there was no reduction in signal intensity, suggesting it was more robust at high antibody concentrations. At the lower end of the titration curve, the Pictor platform appeared to detect signals at lower mAb concentrations than the IDvet assay, suggestive of improved sensitivity and an enhanced dynamic range.

To determine the LoD, twenty replicate measurements were conducted at mAb concentrations near the LoB and assayed over three independent days (Figures 2B–D). The LoD was defined as the lowest concentration at which ≥ 95% of replicates yielded a signal above the LoB across all assay runs. The Pictor PictVet™ ELISA had LoDs of 0.97 ng/mL for the MilA antigen (Figure 2B) and 0.48 ng/mL for the K310 antigen (Figure 2C). In contrast, the IDvet ELISA had a higher LoD of 1.95 ng/mL (for the MilA monoclonal antibody) (Figure 2C). These results indicate that the Pictor PictVet™ Multiplex ELISA may have superior analytical sensitivity, an extended dynamic range, and greater assay stability at both high and low antibody concentrations, supporting its utility as a robust and reliable diagnostic platform for detecting *Mycoplasma bovis*-specific antibodies in cattle.

## 4 Discussion

In this study, we optimized and developed the Pictor PictVet™ *Mycoplasma bovis* IgG Multiplex ELISA, a novel diagnostic assay designed for the sensitive and specific detection of *M. bovis*-specific antibodies in bovine serum. This assay was developed to meet the need for highly sensitive and specific diagnostic tools in support of surveillance and eradication programmes, such as New Zealand’s National *M. bovis* Eradication Programme. To achieve this, we incorporated two immunodominant *M. bovis* antigens—MilA and K310—into a proprietary multiplex format, distinguishing our assay from currently available serological tests, which rely on monoplex ELISA systems.

Compared to the design of other commercial assays such as the ID Screen® *Mycoplasma bovis* Indirect ELISA (IDvet, France) and the BIO K302 ELISA (Bio-X Diagnostics, Belgium), the Pictor PictVet™ ELISA offers notable conceptual advantages in terms of antigen multiplexing and potential for improved diagnostic performance. Most commercial ELISA kits use a single antigen in a conventional 96-well format, whereas the Pictor PictVet™ Multiplex ELISA multiplexes two distinct recombinant antigens within a single well using Pictor’s proprietary PictArray™. This design enables simultaneous detection of antibodies targeting different proteins of *M. bovis*, thereby improving sensitivity.

In our assay development, the Pictor PictVet™ Multiplex ELISA achieved a PPA of 100% and NPA of 98.4%, using the IDvet ELISA as a comparator test. While the absence of a true gold standard makes direct comparison of assays challenging, previously published studies have reported variability in the sensitivity of the IDvet ELISA, with performance influenced by factors such as the characteristics of the sample cohort and study design (11, 21, 22). We compared the capacity of the two assays to detect a monoclonal antibody against the *M. bovis* MilA protein by evaluating their LoDs. Our results showed that the Pictor PictVet™ ELISA had a lower LoD than the IDvet ELISA - 0.97 ng/mL compared to 1.95 ng/mL. The LOD for the Pictor PictVet™ ELISA for a mAb against the K310 protein was also low - 0.48 ng/mL. There was no evidence of a prozone effect in the Pictor PictVet™ platform, even at high antibody concentration, an effect that is occasionally observed in traditional ELISA, demonstrating the robustness and reliability of our multiplex system. Several technical factors may contribute to this observation. First, Pictor employs microdroplet printing to deposit antigens as discrete spots within each well, allowing precise control over local antigen density. This spatial separation may help preserve epitope accessibility and reduce the risk of antigen saturation. In addition, the use of optimized buffer systems during both antigen spotting and assay procedures may enhance antigen stability and minimise non-specific interactions. These conditions could reduce competitive interference between excess antibodies and antigen-binding sites, a known contributor to the prozone effect. Finally, the high analytical sensitivity of the Pictor platform permits the use of higher sample dilutions, which may further lower the risk of signal suppression at elevated antibody titres. While these factors may explain the absence of a prozone effect, further mechanistic studies would be required to confirm their individual contributions.

The implementation of a multiplex strategy combining MilA and K310 significantly enhanced overall assay performance. Individually, both antigens yielded high sensitivity and specificity, but combining the results obtained with both antigens achieved a PPA of 100% and a NPA of 98.4%, with an overall agreement of 98.9%, outperforming either antigen alone. This complementarity supports the hypothesis that incorporating multiple immunodominant antigens can improve detection of immune responses against a pathogen, particularly in animals at different stages of infection or with differing antibody profiles.

Theoretical and practical benefits of multiplexing in ELISA-based diagnostics are well established. Firstly, it enables the detection of a broader range of antibody responses, enhancing sensitivity and reducing the risk of false negatives, especially in subclinical or early infections (23, 24). Secondly, by interpreting the outcome by incorporating results obtained with multiple antigens, specificity can also be improved, minimizing the impact of non-specific reactivity (25). Operationally, multiplex assays reduce reagent use, labour, and cost per sample, which is particularly advantageous in high-throughput settings like national surveillance programmes. Furthermore, multiplex platforms, such as the Pictor PictVet™ system, can be adapted to include additional targets, facilitating surveillance of complex diseases, like the bovine respiratory disease complex (BRDC), and also simultaneous surveillance for multiple pathogens. This extensibility enhances the overall diagnostic utility of the platform while minimizing workflow complexity and sample handling.

Our findings are consistent with recent studies highlighting the promise of multiplex immunoassays in veterinary diagnostics (26–29). For example, Moens et al. (30) reported the development of a Luminex-based multiplex immunoassay for detecting antibodies against multiple *Mycobacterium bovis* antigens, demonstrating the utility of multiplexing for comprehensive serological surveillance. Similarly, bead-based and printed-array platforms have been used for simultaneous detection of antibodies against multiple bovine pathogens, optimizing resource use and improving diagnostic workflows. Despite these advances, no commercial multiplex serological assay is currently available for the simultaneous detection of antibodies against multiple *Mycoplasma bovis*-specific antigens. This lack of multiplex capability represents a critical gap in current diagnostic tools, especially given the complex immunological responses associated with *M. bovis* infections and the pressing need for scalable, high-performance assays in eradication efforts. The development of the Pictor PictVet™ Multiplex ELISA directly addresses this gap by offering a highly sensitive, specific, and operationally efficient platform for the detection of *M. bovis*-specific antibodies. Its capacity to test simultaneously for antibodies against multiple antigens within a single well has the potential not only to enhance diagnostic accuracy, but also to reduce assay time, labour, and cost - key attributes for deployment in large-scale surveillance and eradication programmes. Furthermore, the platform’s flexibility allows for future expansion to include antigens from additional pathogens, positioning it as a next-generation solution for surveillance in bovine health management. Collectively, these advantages underscore the potential of the Pictor PictVet™ platform to become a dominant diagnostic tool in the global fight against *M. bovis* and other pathogens of cattle.

## Data Availability

The original contributions presented in the study are included in the article/supplementary material, further inquiries can be directed to the corresponding author.

## References

[1] Burki S Frey J Pilo P. Virulence, persistence and dissemination of *Mycoplasma bovis*. Vet Microbiol. (2015) 179:15–22. doi: 10.1016/j.vetmic.2015.02.024, PMID: 25824130

[2] Nicholas RA Ayling RD. *Mycoplasma bovis*: disease, diagnosis, and control. Res Vet Sci. (2003) 74:105–12. doi: 10.1016/S0034-5288(02)00155-8, PMID: 12589733

[3] Caswell JL Archambault M. *Mycoplasma bovis* pneumonia in cattle. Anim Health Res Rev. (2007) 8:161–86. doi: 10.1017/S1466252307001351, PMID: 18218159

[4] Maeda T Shibahara T Kimura K Wada Y Sato K Imada Y. *Mycoplasma bovis-*associated suppurative otitis media and pneumonia in bull calves. J Comp Pathol. (2003) 129:100–10. doi: 10.1016/S0021-9975(03)00009-4 12921715

[5] Alberti A Addis MF Chessa B Cubeddu T Profiti M Rosati S. Molecular and antigenic characterization of a Mycoplasma bovis strain causing an outbreak of infectious keratoconjunctivitis. J Vet Diagn Invest. (2006) 18:41–51. doi: 10.1177/104063870601800106, PMID: 16566256

[6] Houlihan MG Veenstra B Christian MK Nicholas R Ayling R. Mastitis and arthritis in two dairy herds caused by Mycoplasma bovis. Vet Rec. (2007) 160:126–7. doi: 10.1136/vr.160.4.126, PMID: 17259455

[7] Hazelton MS Morton JM Parker AM Sheehy PA Bosward KL Malmo J. Whole dairy herd sampling to detect subclinical intramammary Mycoplasma bovis infection after clinical mastitis outbreaks. Vet Microbiol. (2020) 244:108662. doi: 10.1016/j.vetmic.2020.108662, PMID: 32402350

[8] Punyapornwithaya V Fox LK Hancock DD Gay JM Alldredge JR. Association between an outbreak strain causing mycoplasma bovis mastitis and its asymptomatic carriage in the herd: a case study from Idaho, USA. Prev Vet Med. (2010) 93:66–70. doi: 10.1016/j.prevetmed.2009.08.008, PMID: 19880206

[9] Ministry for Primary Industires. (2017). Mycoplasma bovis. New Zealand Government.

[10] Biddle MK Fox LK Hancock DD. Patterns of mycoplasma shedding in the milk of dairy cows with intramammary mycoplasma infection. J Am Vet Med Assoc. (2003) 223:1163–6. doi: 10.2460/javma.2003.223.1163, PMID: 14584748

[11] Andersson AM Aspán A Wisselink HJ Smid B Ridley A Pelkonen S. A European inter-laboratory trial to evaluate the performance of three serological methods for diagnosis of Mycoplasma bovis infection in cattle using latent class analysis. BMC Vet Res. (2019) 15:369. doi: 10.1186/s12917-019-2117-0, PMID: 31653217 PMC6814985

[12] Salgadu A Cheung A Schibrowski ML Wawegama NK Mahony TJ Stevenson MA. Bayesian latent class analysis to estimate the optimal cut-off for the MilA ELISA for the detection of Mycoplasma bovis antibodies in sera, accounting for repeated measures. Prev Vet Med. (2022) 205:105694. doi: 10.1016/j.prevetmed.2022.105694, PMID: 35751981

[13] Salgadu A Firestone SM Watt A Thilakarathne DS Condello AK Siu D. Evaluation of the MilA ELISA for the diagnosis of herd infection with Mycoplasma bovis using bulk tank milk and estimation of the prevalence of M. bovis in Australia. Vet Microbiol. (2022) 270:109454. doi: 10.1016/j.vetmic.2022.109454, PMID: 35597149

[14] Vähänikkilä N Pohjanvirta T Haapala V Simojoki H Soveri T Browning GF. Characterisation of the course of Mycoplasma bovis infection in naturally infected dairy herds. Vet Microbiol. (2019) 231:107–15. doi: 10.1016/j.vetmic.2019.03.007 30955796

[15] Petersen MB Wawegama NK Denwood M Markham PF Browning GF Nielsen LR. Mycoplasma bovis antibody dynamics in naturally exposed dairy calves according to two diagnostic tests. BMC Vet Res. (2018) 14:258. doi: 10.1186/s12917-018-1574-1, PMID: 30165859 PMC6117878

[16] Schibrowski ML Barnes TS Wawegama NK Vance ME Markham PF Mansell PD. The performance of three immune assays to assess the serological status of cattle experimentally exposed to Mycoplasma bovis. Vet Sci. (2018) 5:27. doi: 10.3390/vetsci5010027 29518043 PMC5876582

[17] Wawegama NK Browning GF Kanci A Marenda MS Markham PF. Development of a recombinant protein-based enzyme-linked immunosorbent assay for diagnosis of Mycoplasma bovis infection in cattle. Clin Vaccine Immunol. (2014) 21:196–202. doi: 10.1128/CVI.00670-13, PMID: 24334686 PMC3910936

[18] Wawegama NK Markham PF Kanci A Schibrowski M Oswin S Barnes TS. Evaluation of an IgG enzyme-linked immunosorbent assay as a serological assay for detection of Mycoplasma bovis infection in feedlot cattle. J Clin Microbiol. (2016) 54:1269–75. doi: 10.1128/JCM.02492-15, PMID: 26912757 PMC4844740

[19] Clinical and Laboratory Standards Institute. Protocols for determination of limits of detection and limits of quantitation, approved guideline. Wayne, Pennsylvania, USA: P.U.C. Wayne (2004).

[20] Armbruster DA Pry T. Limit of blank, limit of detection and limit of quantitation. Clin Biochem Rev. (2008) 29 Suppl 1:S49–52. 18852857 PMC2556583

[21] Marquetoux N Vignes M Burroughs A Sumner E Sawford K Jones G. Evaluation of the accuracy of the IDvet serological test for Mycoplasma bovis infection in cattle using latent class analysis of paired serum ELISA and quantitative real-time PCR on tonsillar swabs sampled at slaughter. PLoS One. (2023) 18:e0285598. doi: 10.1371/journal.pone.0285598, PMID: 37167206 PMC10174590

[22] Veldhuis A Aalberts M Penterman P Wever P van Schaik G. Bayesian diagnostic test evaluation and true prevalence estimation of mycoplasma bovis in dairy herds. Prev Vet Med. (2023) 216:105946. doi: 10.1016/j.prevetmed.2023.105946, PMID: 37235906

[23] le D Bézille P Calavas D Poumarat F Brank M Citti C. Serological prevalence of Mycoplasma bovis infection in suckling beef cattle in France. Vet Rec. (2002) 150:268–73. doi: 10.1136/vr.150.9.268, PMID: 11918048

[24] Le Grand D Solsona M Rosengarten R Poumarat. Adaptive surface antigen variation in Mycoplasma bovis to the host immune response. FEMS Microbiol Lett. (1996) 144:267–75. doi: 10.1111/j.1574-6968.1996.tb08540.x, PMID: 8900072

[25] Salih BA Rosenbusch RF. Cross-reactive proteins among eight bovine mycoplasmas detected by monoclonal antibodies. Comp Immunol Microbiol Infect Dis. (2001) 24:103–11. doi: 10.1016/S0147-9571(00)00020-5, PMID: 11247043

[26] Clavijo A Hole K Li M Collignon B. Simultaneous detection of antibodies to foot-and-mouth disease non-structural proteins 3ABC, 3D, 3A and 3B by a multiplexed Luminex assay to differentiate infected from vaccinated cattle. Vaccine. (2006) 24:1693–704. doi: 10.1016/j.vaccine.2005.09.057, PMID: 16260073

[27] Nájera-Rivera HD Rodríguez-Cortez AD Anaya-Santillán MG Díaz-Aparicio E Ramos-Rodríguez AV Siliceo-Cantero IJ. Multiplex assay for the simultaneous detection of antibodies against small ruminant lentivirus, Mycobacterium avium subsp. paratuberculosis, and Brucella melitensis in goats. Vet World. (2023) 16:704–10. doi: 10.14202/vetworld.2023.704-710 37235146 PMC10206977

[28] Rodriguez A Alonso-Morales RA Lassala A Rangel P L Ramírez-Andoney V Gutierrez CG. Development and validation of a pentaplex assay for the identification of antibodies against common viral diseases in cattle. Access Microbiol. (2023) 5. doi: 10.1099/acmi.0.000511.v3, PMID: 37970075 PMC10634487

[29] Germeraad E Achterberg R Venema S Post J de Leeuw O Koch G. The development of a multiplex serological assay for avian influenza based on Luminex technology. Methods. (2019) 158:54–60. doi: 10.1016/j.ymeth.2019.01.012, PMID: 30707951

[30] Moens C Filée P Boes A Alie C Dufrasne F André E. Identification of new Mycobacterium bovis antigens and development of a multiplexed serological bead-immunoassay for the diagnosis of bovine tuberculosis in cattle. PLoS One. (2023) 18:e0292590. doi: 10.1371/journal.pone.0292590, PMID: 37812634 PMC10561873

